# Mechanism and Experimental Verification of *Luteolin* for the Treatment of Osteoporosis Based on Network Pharmacology

**DOI:** 10.3389/fendo.2022.866641

**Published:** 2022-03-08

**Authors:** Guihong Liang, Jinlong Zhao, Yaoxing Dou, Yuan Yang, Di Zhao, Zhanpeng Zhou, Rui Zhang, Weiyi Yang, Lingfeng Zeng

**Affiliations:** ^1^ The 2nd Affiliated Hospital of Guangzhou University of Chinese Medicine, Guangzhou, China; ^2^ The Second Clinical Medical College of Guangzhou University of Chinese Medicine, Guangzhou, China; ^3^ School of Pharmaceutical Sciences, Guangzhou University of Chinese Medicine, Guangzhou, China

**Keywords:** Traditional Chinese medicine, luteolin, osteoporosis, network pharmacology, signalling pathway, experimental verification

## Abstract

**Purpose:**

To explore the molecular mechanism of *luteolin* in the treatment of osteoporosis (OP) by network pharmacological prediction and experimentation.

**Methods:**

The target proteins of *luteolin* were obtained with the Traditional Chinese Medicine Systems Pharmacology Database and Analysis Platform (TCMSP). OP-related proteins were extracted from the Comparative Toxicogenomics Database (CTD) and GeneCards and DisGeNET databases. We imported the common protein targets of *luteolin* and OP into the STRING database to obtain the relationships between the targets. The common target proteins of *luteolin* and OP were assessed by KEGG and GO enrichment analyses with the DAVID database. Animal experiments were conducted to verify the effect of *luteolin* on bone mineral density in ovariectomised (OVX) rats. Finally, the effects of *luteolin* on key signalling pathways were verified by cell experiments *in vitro*.

**Results:**

Forty-four targets of *luteolin* involved in the treatment of OP, including key target proteins such as TP53, AKT1, HSP90AA1, JUN, RELA, CASP3, and MAPK1, were screened. KEGG enrichment analysis found that *luteolin* inhibits OP by regulating the PI3K-Akt, TNF, oestrogen and p53 signalling pathways. The results of animal experiments showed that bone mass in the low-dose *luteolin* group (*Luteolin*-L group, 10 mg/kg), high-dose *luteolin* group (*Luteolin*-H group, 50 mg/kg) and positive drug group was significantly higher than that in the OVX group (P<0.05). Western blot (WB) analysis showed that the protein expression levels of Collagen I, Osteopontin and RUNX2 in bone marrow mesenchymal stem cells (BMSCs) cultured with 0.5, 1 and 5 μM *luteolin* for 48 h were significantly higher than those in the dimethyl sulfoxide (DMSO) group (P<0.05). *In vitro* cell experiments showed that the p-PI3K/PI3K and p-Akt/Akt expression ratios in BMSCs cultured with 0.5, 1 and 5 μM *luteolin* for 48 h were also significantly higher than those in the DMSO group (P<0.05).

**Conclusions:**

*Luteolin* has multitarget and multichannel effects in the treatment of OP. *Luteolin* could reduce bone loss in OVX rats, which may be due to its ability to promote the osteogenic differentiation of BMSCs by regulating the activity of the PI3K-Akt signalling pathway.

## Introduction

Osteoporosis (OP) is a systemic bone disease involving decreased bone density and bone quality induced by human ageing, menopause and other factors, and the main characteristics of this disease are the destruction of bone microstructure and an increase in bone brittleness (1, 2). With the ageing of the global population, OP and its complications will become a major public global health problem (3, 4). It is predicted that by 2050, the medical expenses for OP-related fractures in China will reach 25.4 billion USD, nearly 30 times greater than those in 2010 (5). At present, OP is mainly treated by drug therapy, physical therapy and exercise therapy (6, 7). Although great progress has been made in the treatment of OP, therapeutic effects still do not meet clinical expectations. First, many newly developed anti-OP drugs are expensive (8), limiting their wide clinical application; in addition, adverse drug reactions (9) eventually affect patient drug compliance.

**Figure 1:**
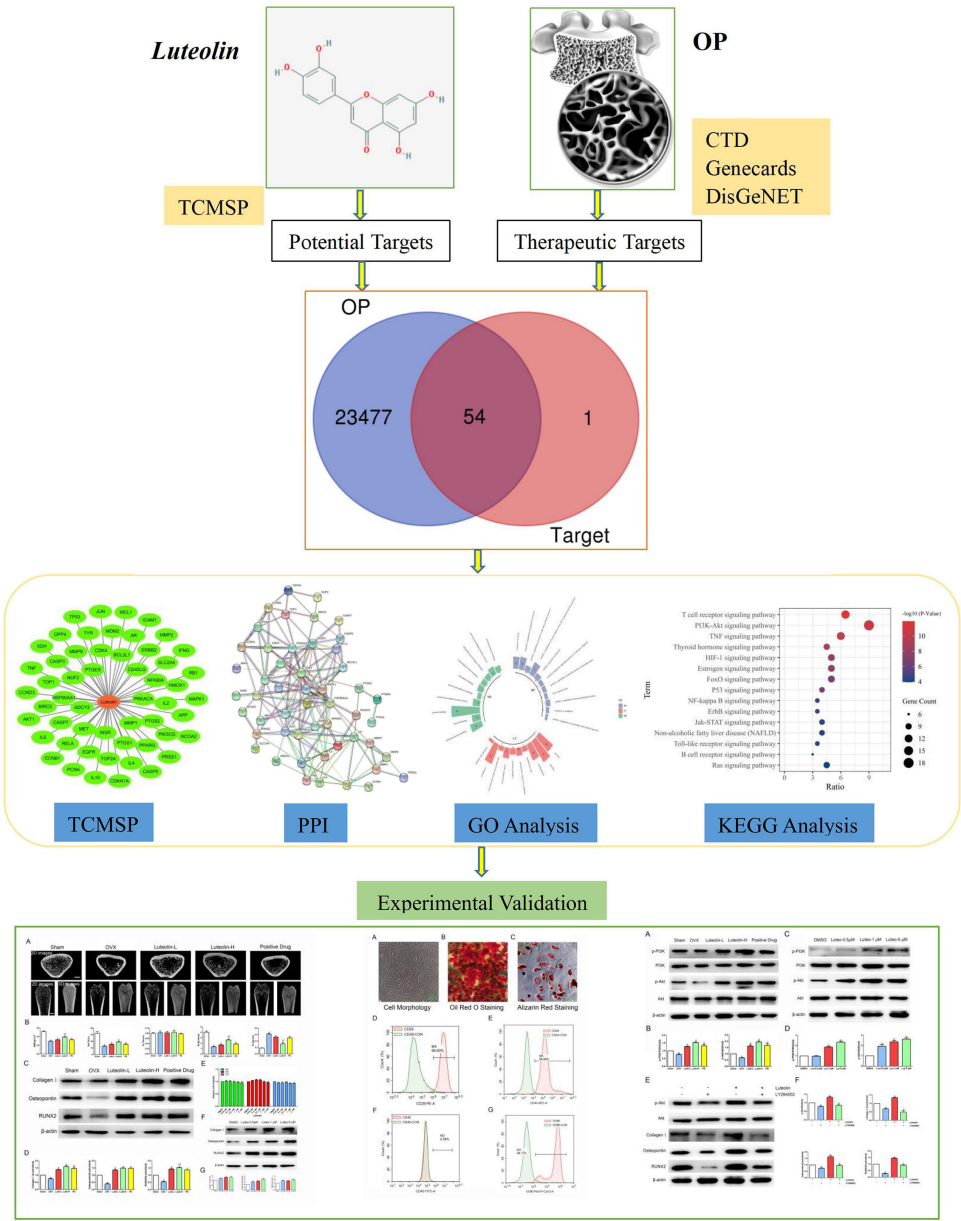
Workflow of Luteolin in treatment of OP.

In the theoretical traditional Chinese medicine system, OP is called “*Gu Wei*”, which refers to osteopenia caused by kidney deficiency. Traditional Chinese medicine has a long history of using herbal medicines to tonify the kidney and replenish *Qi* for the prevention and treatment of “*Gu Wei*”. In the application of herbal traditional Chinese medicines, many kidney-tonifying herbs contain flavonoids or flavonoid monomers (10). *Luteolin* is a natural flavonoid found in various medicinal materials, vegetables and fruits, such as *honeysuckle*, *Schizonepeta tenuifolia* and *Perilla*, in its glycosylated form (11). Many studies have shown that flavonoids, such as *icariin*, *naringin* and *soybean isoflavones*, can inhibit OP through a variety of pathways and targets (10, 12–14). *Luteolin*, a flavonoid monomer, may also have great potential for the treatment of OP. Studies have focused on the mechanism of *luteolin* in the treatment of OP, but clarification of this mechanism is still needed (15, 16). This study aimed to screen the targets of *luteolin* in the treatment of OP through network pharmacology to study the network relationship among the drug, its targets and related signalling pathways. Some key targets were experimentally verified, providing a scientific basis for the mechanism of *luteolin* in the treatment of OP and drug development. A flow chart describing this study is shown in **Figure 1**.

## Materials and Methods

### Network Pharmacology

#### Obtaining Potential Targets of *Luteolin*

The Traditional Chinese Medicine Systems Pharmacology Database and Analysis Platform (TCMSP) (https://tcmspw.com/tcmsp.php) was used to obtain the targets of *luteolin*. Human genes were identified from the target information with the UniProt database (https://www.UniProt.org/).

#### Acquisition of OP Target Genes

OP-related genes were obtained from the Comparative Toxicogenomics Database (CTD) (http://ctdbase.org/) and GeneCards (https://www.genecards.org/) and DisGeNET (http://www.disgenet.org/web/DisGeNET/menu/home) databases with “osteoporosis” as the keyword. The target genes of *luteolin* and OP were introduced into Venny 2.1 to screen genes related to the use of *luteolin* in the treatment of OP.

#### Construction of a Protein–Protein Interaction (PPI) Network and Screening of Core Targets

Common target genes of *luteolin* and OP were imported into the STRING online database (https://string-db.org) for PPI analysis. The species in the database was set to “*Homo sapiens*”, and the screening confidence was ≥ 0.90. With the above parameters, a PPI network interaction diagram was obtained, and the core gene was then obtained based on the number of node connections.

#### Construction of a “*Luteolin*-Target” Network Diagram

The targets of *luteolin* in OP were visualized with Cytoscape 3.7.1.

#### Gene Function and Biological Pathway Enrichment Analyses

The target genes of *luteolin* in the treatment of OP were uploaded to the DAVID data analysis platform for KEGG pathway enrichment and GO biological process enrichment analyses. The species was limited to “*Homo sapiens*” in the database. Pathways and biological processes for which *P* < 0.05 were extracted.

### Experimental Verification

#### Animals and Experimental Groups

A total of 20 SPF-grade female SD rats aged 1.5 months with a body weight of (200 ± 10) g were used. The above rats were purchased from the *Experimental Animal Center of Southern Medical University*. The animal certificate number is 44002100030321. The twenty rats were raised in an SPF environment at the *Experimental Animal Center of Guangdong College of Traditional Chinese Medicine*. The experimental protocol was implemented after approval by the Animal Ethics Committee of the *Guangdong Hospital of Traditional Chinese Medicine* (animal experimental ethics approval No. 2021028). Relevant experimental procedures were implemented in accordance with regulations related to *the administration of experimental animals* approved by the State Council of the People’s Republic of China.

#### Construction of an OP Rat Model of Bilateral Ovariectomy and Drug Treatment

Twenty rats were divided into five groups (4 rats in each group), namely, the sham group, ovariectomised (OVX) group, low-dose *luteolin* group (*Luteolin*-L group), high-dose *luteolin* group (*Luteolin*-H) and positive drug group. After isoflurane was used to anaesthetise the rats, the rats in all groups except the sham group underwent surgery on both ovaries. For rats in the sham group, the bilateral ovaries were separated, and some adipose tissue around the ovaries was removed. Drug gavage was started 4 weeks after the operation, and *luteolin* was dissolved in carboxymethylcellulose sodium (CMC-na, C304951, Aladdin, Shanghai, China) to prepare a suspension. Alendronate sodium and vitamin D3 tablets (J201440144, Merck Sharp & Dohme Ltd., UK) were dissolved in normal saline. Experimental studies have proved that [16], *luteolin* at 25, 50, and 100 mg/kg doses can alleviate bone loss associated with glucocorticoid-induced osteoporosis induced by gavage in SD rats over 2 months. Based on the principle of reduction and optimisation in the “3R principle” of animal experiments, effective experimental data are obtained on the premise of using as few animals as possible. In this experiment, the bone mass loss of the OP model constructed by the OVX method progressed slowly. Therefore, a high dose of 50 mg/kg was selected to observe the effect of luteolin on the bone mass of OVX rats, and a lower dose of luteolin (10 mg/kg) was used to further explore whether a lower dose of luteolin can also increase the bone mass of OVX rats, representing a safer dose choice for the future treatment of OP with luteolin. Rats in the Luteolin-L group (10 mg/kg body weight) and Luteolin-H group (50 mg/kg body weight) underwent gavage once a day for 8 weeks. Rats in the positive drug group underwent gavage with alendronate sodium and vitamin D3 tablets (6.3 mg/kg) once a week for 8 weeks. Rats in the other two groups were given the same dose of drug dissolved in solvent by gavage. After 8 weeks of gavage, the rats were anaesthetised and euthanised, and the long bones of the limbs were collected for further analysis.

#### Micro-CT Analysis

After 4% paraformaldehyde fixation, a micro-CT scanner (ZKKS-MCT-Sharp, Guangzhou, China) was used to scan and analyse the morphology of the femur. During scanning, the femur was fixed in the fixator along the long axis. The scanning voltage was set to 70 kV, and the current was set to 100 μA. The power was 7 W, 4 frames were superimposed, the angle gain was 0.72 degrees, the scanning was carried out by rotating for one cycle, and the scanning layer was 15 μm thick. ZKKS-MicroCT 4.1 software was used to analyse the bone morphological parameters of the femoral metaphysis.

#### Extraction, Culture, and Identification of Rat Primary BMSCs

Bone marrow fluid was collected from the rat long shaft, and rat BMSCs were extracted by whole-bone-marrow cell culture. The cells were cultured in OriCell^®^ rat bone marrow mesenchymal stem cell (BMSC) complete medium. Expression of the cell surface markers CD29, CD44, CD45 and CD90 was detected by flow cytometry. Osteogenic differentiation of the BMSCs was induced by rat BMSCs with an Osteogenic Differentiation Medium Kit (batch No. RASMX-90021, Saiye (Suzhou) Biotechnology Co., Ltd., Suzhou, China). Cell mineralisation was observed by Alizarin red staining. Adipogenic differentiation of the BMSCs was induced by rat BMSCs with an Adipogenic Differentiation Medium kit (batch No. RASMX-90031, Saiye (Suzhou) Biotechnology Co., Ltd., Suzhou, China). Fat droplets in the cells were observed by oil red O staining.

#### Cell Viability Assay

BMSCs were plated at a density of 2.5 × 10^3^ cells/well in a 96-well plate. After culturing the cells for 24 h, 0.05 μM, 0.1 μM, or 5 μM luteolin (S2320, Selleck, USA) was added, and the cells were then incubated for a further 24, 48, or 72 h. Dimethyl sulfoxide (DMSO, D2650, Sigma, USA) was used to treat the control group. At the end of luteolin treatment, 10 μL of reagent from a Cell Counting Kit-8 (CCK-8, C0038, Beyotime, China) was added to each well, and the cells were incubated for 2 h at 37°C. The optical density (OD) at 450 nm was determined using a microplate reader (M1000 Pro, Tecan, Switzerland); four independent experiments were carried out.

#### Western Blot (WB) Analysis

After BMSCs were treated with the corresponding drugs, cell lysis buffer for WB and IP (P0013, Beyotime Biotechnology, China) was used to collect the total protein from the cells, and a bicinchoninic acid (BCA) quantification kit (P0012, Beyotime Biotechnology, China) was used to detect the protein concentration. A total of 20 μg of total protein was subjected to SDS–PAGE electrophoresis and then transferred to PVDF membranes. The membranes were blocked with 5% skimmed milk at room temperature for 1 h, and the following diluted antibodies were then added: anti-collagen I (1:1000, ab260043, Abcam), anti-osteopontin (1:1000, ab63856, Abcam), anti-RUNX2 (1:1000, 12556, CST), anti-β-actin (1:2000, 3700, CST), anti-PI3K (1:1000, 3358, CST), anti-Akt (1:1000, 4691, CST), anti-phospho-Akt (Ser473) (1:1000, 4058, CST), and anti-PI3K p85 (1:1000, ab182651, Abcam). The PVDF membranes were washed 3 times with 1× TBST. Finally, the samples were incubated in a refrigerator at 4°C overnight. HRP-conjugated goat anti-rabbit or anti-mouse antibodies (7074, 7076, CST, USA) were added and incubated at room temperature for 1 h. Finally, the membranes were incubated with ECL reagent (WBKLS0100, Merck, Germany) and scanned with a chemiluminescence system (Bio–Rad, USA).

#### Data Analysis

The data were analysed using one-way analysis of variance (ANOVA) followed by Tukey’s multiple comparison test (GraphPad Prism version 5.0 for Windows).

## Results

### Common Targets of *Luteolin* and OP

**Figure 2:**
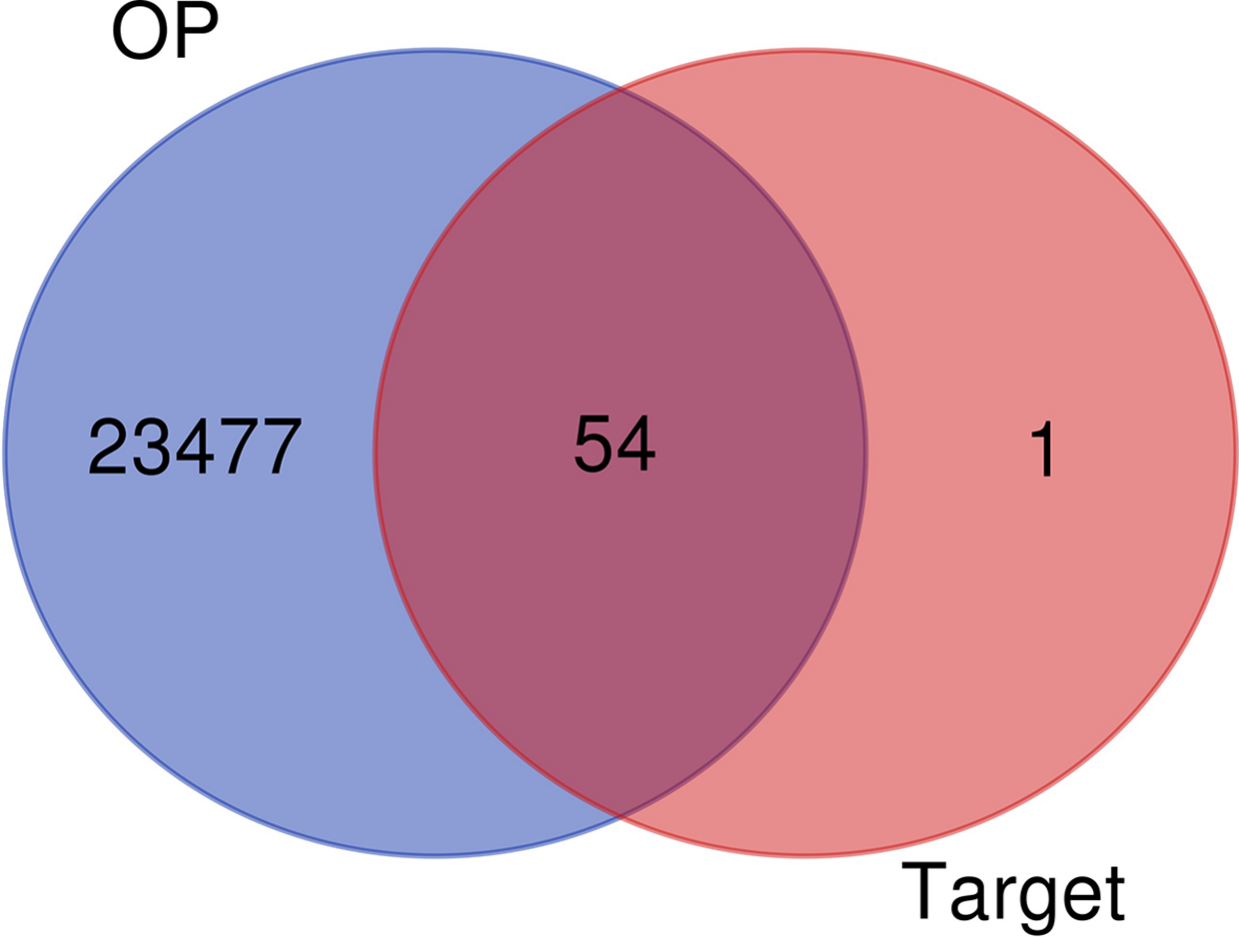
Venn diagram of Luteolin targets and OP-related targets.

A total of 57 *luteolin* targets (including 2 invalid targets) were retrieved with the TCMSP. We obtained 36180 gene targets of OP from the CTD, 1098 gene targets from the DisGeNET database and 2916 gene targets from the GeneCards database. After removing the duplicates, 23531 OP gene targets were obtained. After mapping the targets of *luteolin* and OP with Venny 2.1, 54 intersecting targets were obtained (**Figure 2**).

### “*Luteolin*-Target” Network Diagram

**Figure 3:**
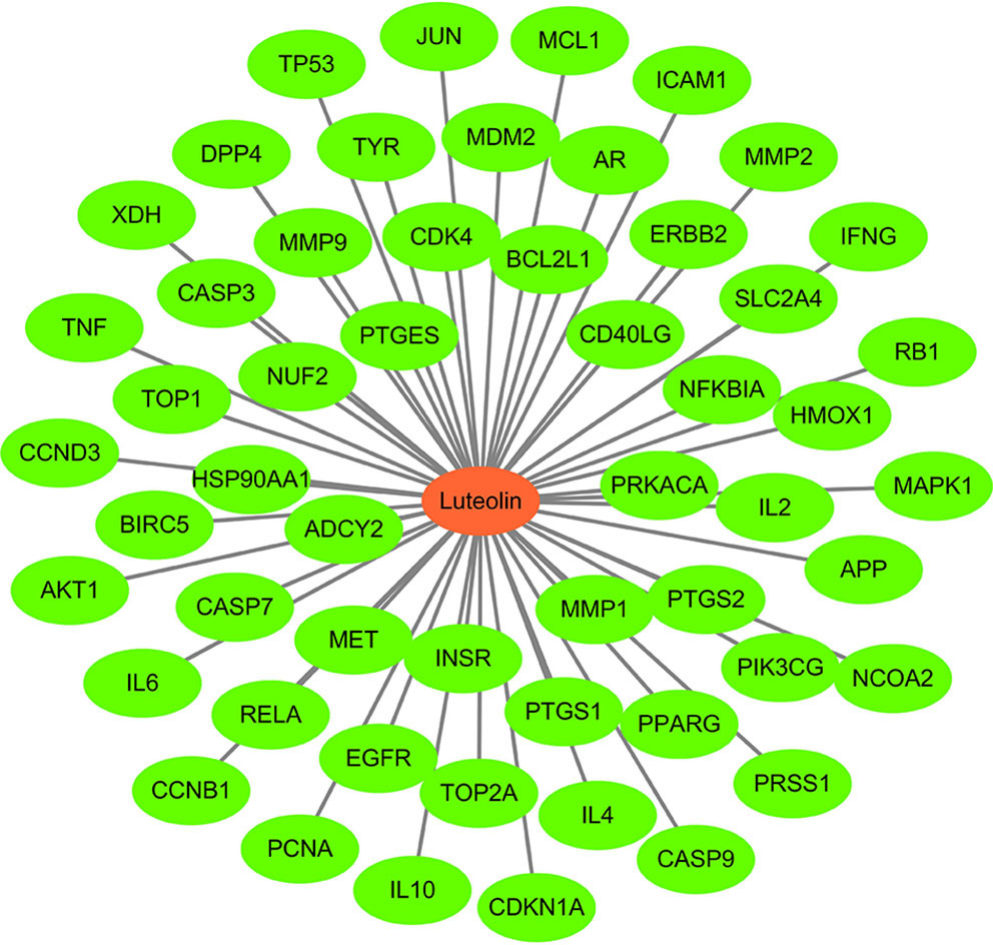
Potential target genes related to Luteolin.

Using Cytoscape 3 7.1 software, a “*luteolin*-target” network diagram was drawn (**Figure 3**). In the figure, the red node is *luteolin*, and each green node is a target.

### PPI Network Construction

**Figure 4:**
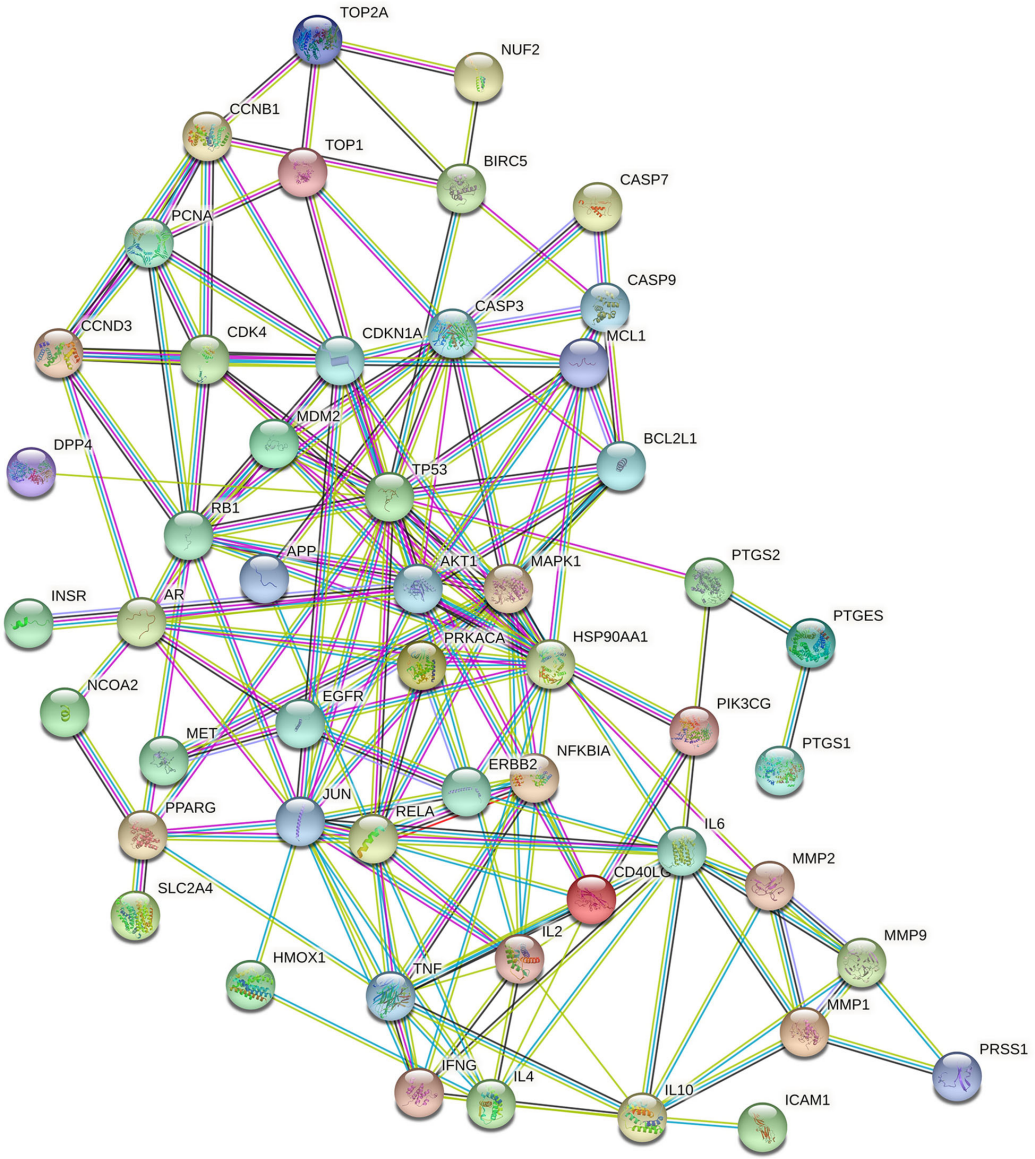
The PPI network of Luteolin.

**Figure 5:**
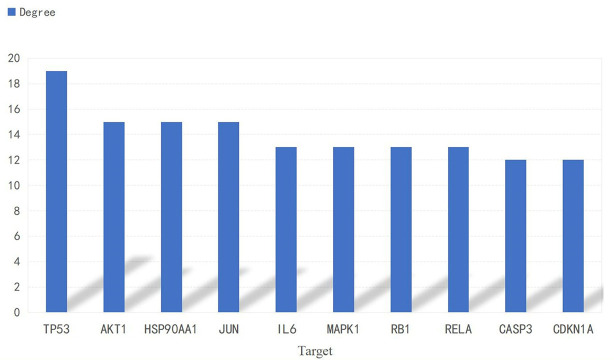
Histogram of the adjacent target number.

A PPI network consisting of common targets was obtained by using the STRING 11.0 data analysis platform with the confidence set to ≥0.90. After hiding the isolated protein, a PPI network diagram was obtained. A total of 54 proteins were obtained (**Figure 4**). The degree value is often used to indicate the importance of network nodes. The larger the degree value is, the more important the node is in the network is. The top 10 most important targets in the PPI network based on degree value were TP53, AKT1, HSP90AA1, JUN, IL6, MAPK1, RELA, RB1, CASP3, and CDKN1A (**Figure 5**).

### Results of GO Enrichment Analysis

**Figure 6:**
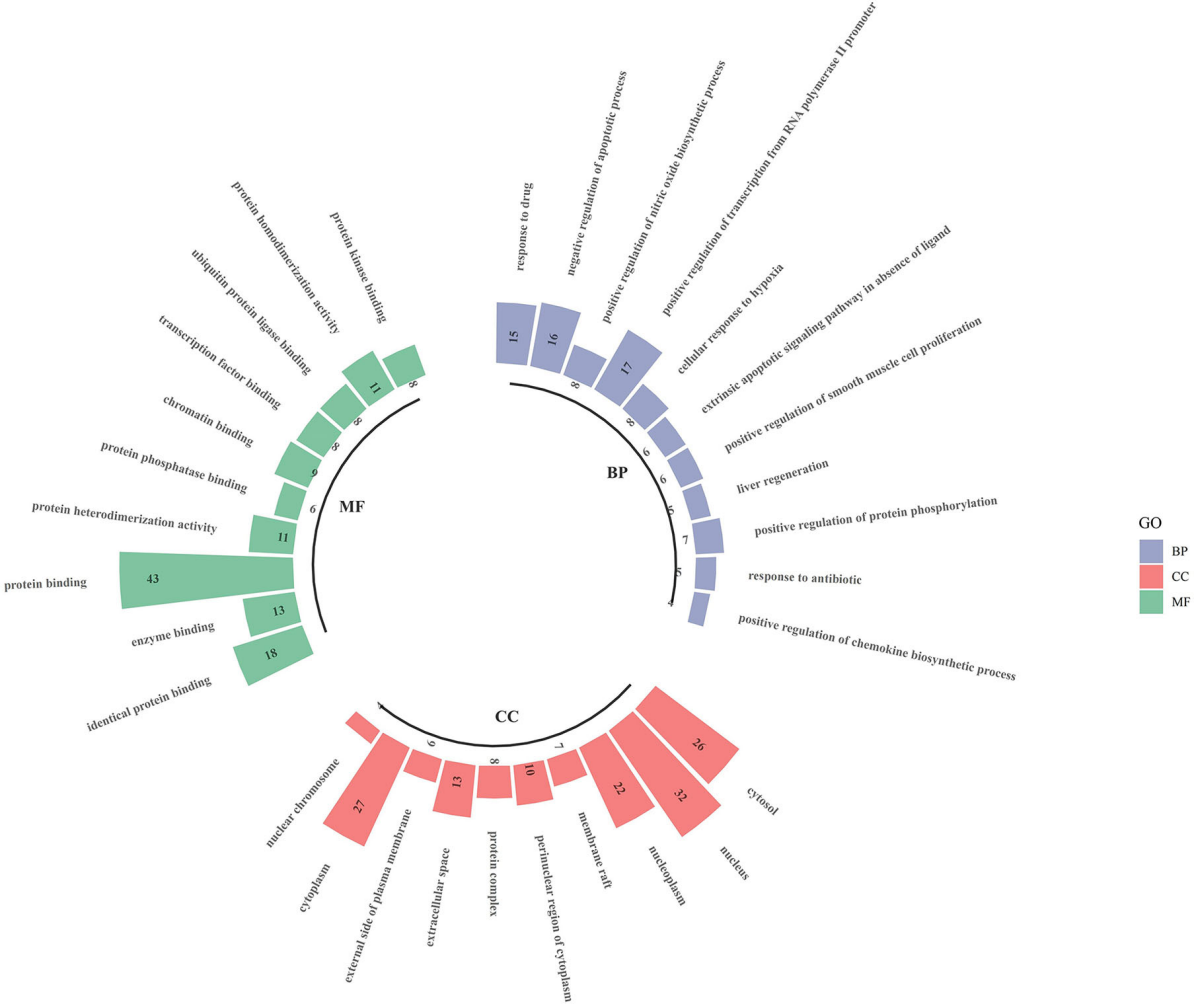
Go enrichment analysis for targets of Luteolin against OP (TOP 10).

The intersecting targets of *luteolin* and OP were analysed for GO functional enrichment with the DAVID data analysis platform, and the top 10 most significantly enriched biological process (BP), cell composition (CC) and molecular function (MF) terms were selected to form a GO enrichment bar graph (**Figure 6**). BP enrichment included the response to drugs, negative regulation of apoptotic processes, positive regulation of nitric oxide biosynthetic processes, and positive regulation of transcription from RNA polymerase II promoter. CC enrichment included the cytosol, nucleus, nucleoplasm, membrane raft, etc. MF enrichment included identical protein binding, enzyme binding, protein binding, protein heterodimerisation activity, and protein phosphatase binding.

### Results of KEGG Enrichment Analysis

**Figure 7:**
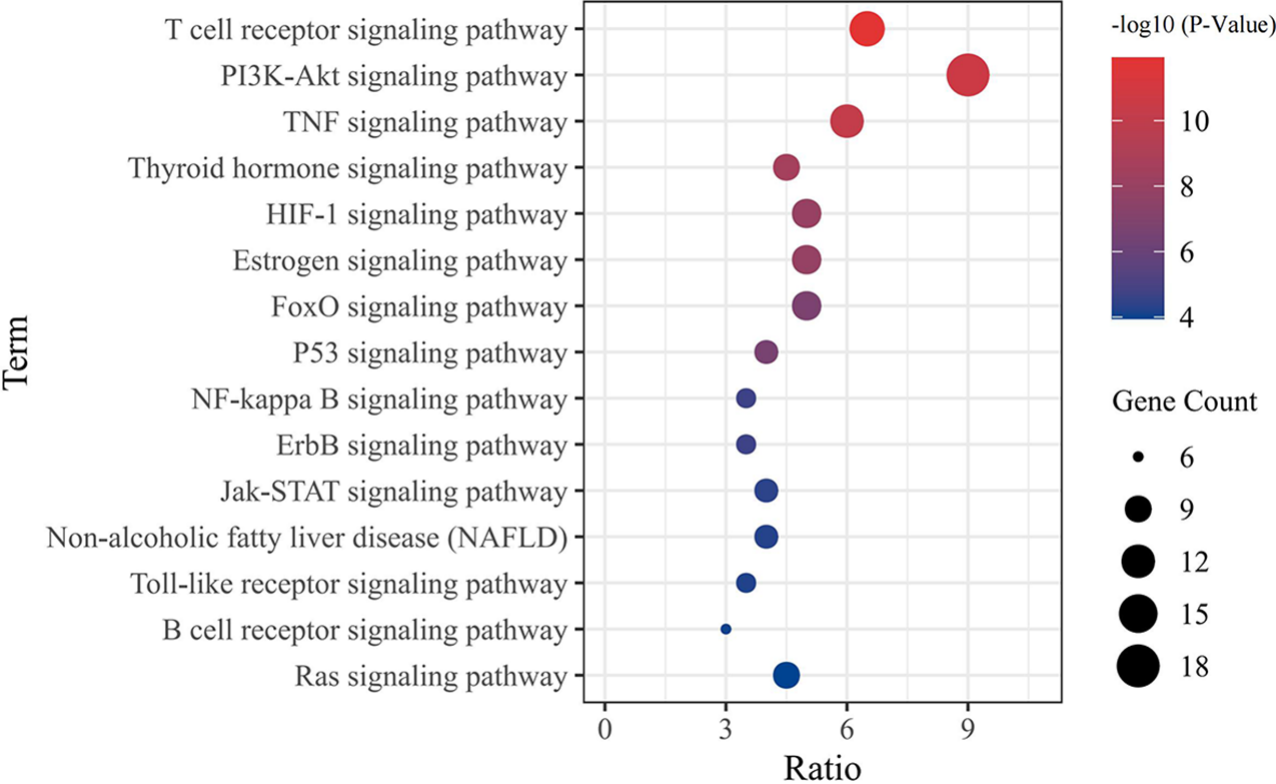
The bubble chart of KEGG pathway analysis (TOP 15).

The enrichment of KEGG pathways in the intersecting targets of *luteolin* and OP were analysed with the DAVID data analysis platform. Ultimately, a total of 109 signalling pathways were obtained. After excluding obviously unrelated disease pathways, such as tumour- and liver disease-related pathways, the top 15 most significantly enriched pathways were selected and used to form a bubble diagram consisting of enriched KEGG signalling pathways (**Figure 7**). The results showed that *luteolin* may inhibit OP by regulating the T cell receiver, PI3K-Akt, TNF, HIF-1, oestrogen, FoxO and p53 signalling pathways.

### *Luteolin* Alleviated Bone Loss in an OP Model by Promoting the Osteogenic Differentiation of BMSCs

**Figure 8:**
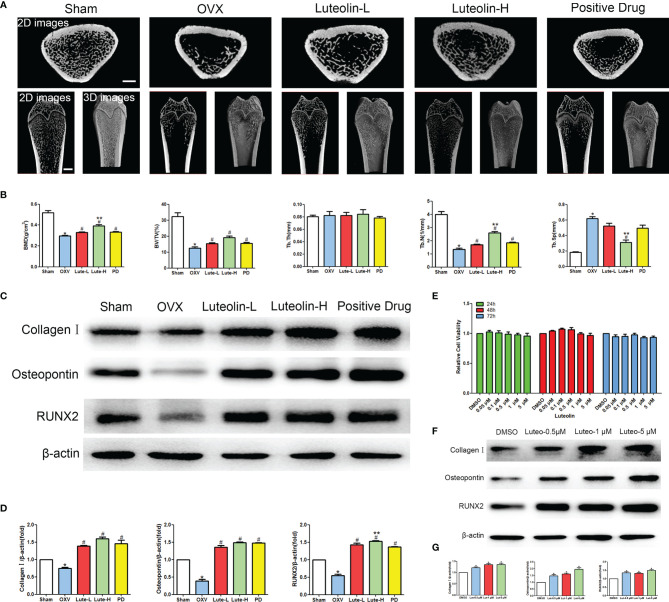
Luteolin alleviated bone loss in OVX rats by promoting the osteogenic differentiation of BMSCs. **(A)** Micro-CT image of the femoral diaphysis (scale bars, 1 mm). **(B)** Quantitative presentation of the femoral microarchitectural parameters BMD, BV/TV, Tb.Th, Tb.N, and Tb.Sp expressed as the mean ± SD from four independent experiments. Compared with the sham group, *p < 0.05; compared with the OVX group, #*p* < 0.05; compared with the DG group, **p < 0.05. **(C)** The expression levels of osteogenesis-related marker proteins (collagen I, osteopontin and RUNX2). **(D)** Comparative analysis of grey values for relevant protein bands; data are expressed as the mean ± SD from three independent experiments. Compared with the sham group, *p < 0.05; compared with the OVX group, #*p* < 0.05; compared with the DG group, **p < 0.05. **(E)** BMSCs were treated with luteolin at increasing concentrations (0.05 μM, 0.1 μM, 0.5 μM, 1 μM, and 5 μM) for 24, 48 and 72 h, after which the OD value was determined by CCK-8 assay. Cell viability is expressed as the mean ± SD from four independent experiments. There was no significant difference. **(F)** BMSCs were treated with luteolin at increasing concentrations (0.5 μM, 1 μM, and 5 μM) for 48 h, after which osteogenesis-related marker protein (collagen I, osteopontin, RUNX2) expression was analysed with WB. **(G)** Comparative analysis of grey values from relevant protein bands; data are expressed as the mean ± SD from three independent experiments. Compared with the DMSO group, *p < 0.05.

**Figure 9:**
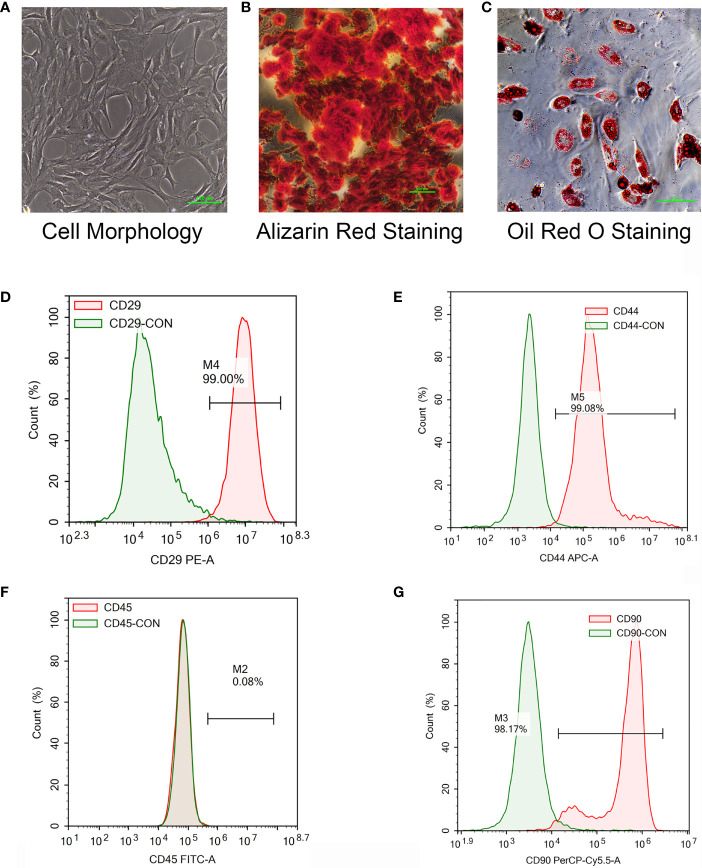
Isolation and culture of rat primary BMSCs, the induction of osteogenic and adipogenic differentiation and the detection of cell surface markers. **(A)** The morphology of primary BMSCs from third-generation rats was observed under a microscope (×40). **(B)** After the osteogenic differentiation of third-generation BMSCs was induced with an osteogenic differentiation-inducing agent (Alizarin red staining), the formation of mineralized nodules was observed under a microscope (×100). **(C)** The adipogenic differentiation of third-generation BMSCs was induced by an adipogenic differentiation-inducing agent, and lipid droplets were enlarged and made rounder by continuous culture with maintenance medium (stained with oil red O) and observed under a microscope (×200). **(D–F)** The primary BMSCs of third-generation rats were collected and stained for CD29 **(D)**, CD44 **(E)**, CD45 **(F)** and CD90 **(G)** for 30 min. Control staining was simultaneously carried out. Fluorescence was detected with a flow cytometer, and the results were analysed.

To verify the mechanism of *luteolin* in the treatment of OP, we administered *luteolin* (low-dose group: 10 mg/kg, high-dose group: 50 mg/kg) to OVX model rats by gavage and used alendronate D3 tablets as the positive control. After 8 weeks of gavage, the femurs were collected from the rats in each group, and the distal femur was scanned by micro-CT. The imaging results showed that bone mass was significantly decreased for rats in the OVX group compared with those in the sham group. However, bone mass was significantly greater for the *Luteolin*-L group, *Luteolin*-H group and positive drug group compared with the OVX group (**Figure 8A**). An analysis of bone morphological parameters showed that the bone mineral density (BMD), relative bone volume fraction (BV/TV) and trabecular number (Tb.N) were significantly lower for the OVX group than for the sham group, but the values of the *Luteolin*-L group, *Luteolin*-H group and positive drug group were significantly higher than those of the OVX group. The BMD and Tb.N values were greater for the *Luteolin*-H group than for the positive drug group. Trabecular separation (Tb.Sp) was significantly greater for the OVX group than for the sham group. Furthermore, the Tb.Sp of the *Luteolin*-H group was smaller than that of the OVX group and smaller than that of the positive drug group. There was no significant difference in trabecular thickness (Tb.Th) of the femur between the groups (**Figure 8B**). We collected long bone marrow from the rats in each group, extracted BMSCs and examined the isolated and cultured primary BMSCs. The results showed that the BMSCs grew normally after separation, and the cells were long and spindle-shaped under an inverted microscope (**Figure 9A**). After osteogenic induction, the results of alizarin red staining suggested the presence of a large number of mineralised nodules stained red (**Figure 9B**). After lipid induction, oil red O staining showed a large number of lipid droplets dyed orange (**Figure 9C**). Through fluorescent flow cytometry analysis of CD29, CD44, CD45 and CD90, the results showed that the CD29, CD44, CD45 and CD90 positivity rates were 99.00%, 99.08%, 0.08% and 98.17%, respectively (**Figures 9D–G**), in line with the biological characteristics of BMSCs. We extracted total protein from the BMSCs in each group and analysed osteogenic differentiation-related protein levels by WB. The results showed that the protein expression levels of collagen I, osteopontin and RUNX2 in BMSCs of the OVX group were significantly lower than those of the sham group, but the values of the *Luteolin*-L group, *Luteolin*-H group and positive drug group were significantly higher than those of the OVX group, and the expression of RUNX2 in the *Luteolin*-H group was also higher than that in the positive drug group (**Figures 8C, D**). To verify the effect of *luteolin* on the osteogenic differentiation of BMSCs, we further analysed the effect of *luteolin* on the viability of BMSCs and the expression of osteogenic differentiation-related proteins through *in vitro* cellular experiments. The results of CCK-8 assays showed that the viability of the BMSCs was not affected after 72 h of culture with *luteolin* at a concentration of 5 μM (**Figure 8E**). The results of WB showed that the protein expression levels of collagen I, osteopontin and RUNX2 in BMSCs cultured with *luteolin* at concentrations of 0.5, 1 and 5 μM were significantly higher than those in the DMSO group (**Figures 8F, G**). The above results show that *luteolin* could reduce bone loss in OVX rats, inhibit the progression of OP, and promote the osteogenic differentiation of BMSCs.

### *Luteolin* Promotes the Osteogenic Differentiation of BMSCs by Activating the PI3K/Akt Signalling Pathway

**Figure 10:**
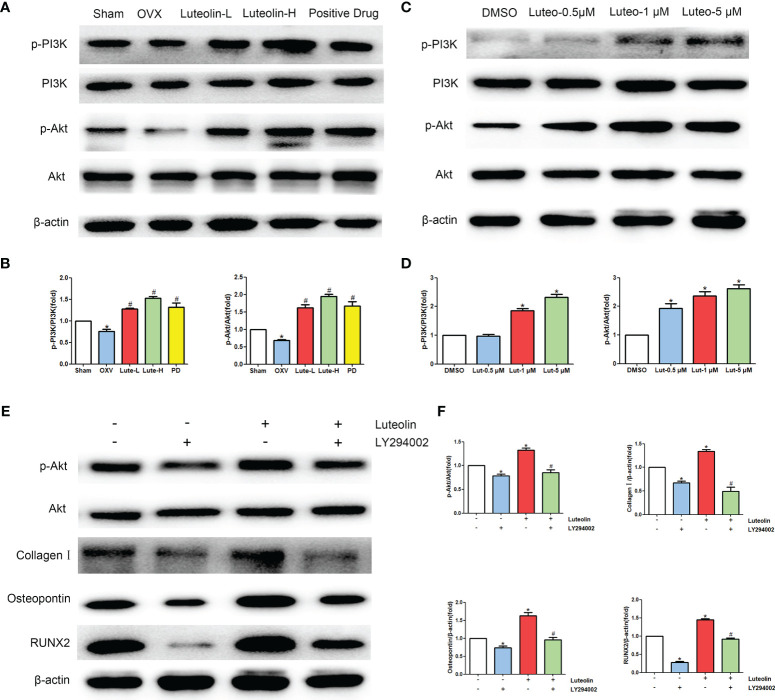
Luteolin promotes the osteogenic differentiation of BMSCs by activating the PI3K/Akt signalling pathway. **(A)** The protein expression of p-PI3K, PI3K, p-Akt and Akt was detected by WB. **(B)** Comparative analysis of grey values from relevant protein bands; data are expressed as the mean ± SD from three independent experiments. Compared with the sham group, *p < 0.05; compared with the OVX group, #*p* < 0.05. **(C)** BMSCs were treated with luteolin at increasing concentrations (0.5 μM, 1 μM, and 5 μM) for 48 h, followed by an analysis of p-PI3K, PI3K, p-Akt and Akt protein expression levels with WB. **(D)** Comparative analysis of grey values from relevant protein bands; data are expressed as the mean ± SD from three independent experiments. Compared with the DMSO group, *p < 0.05. **(E)** BMSCs were incubated with or without 20 μM LY294002 (S1105, Selleck, USA) and 5 μM luteolin for 48 h, followed by an analysis of collagen I, osteopontin, RUNX2, p-Akt and Akt protein expression levels by WB. **(F)** Comparative analysis of grey values from relevant protein bands; data are expressed as the mean ± SD from three independent experiments. Compared with the DMSO group, *p < 0.05; compared with the luteolin only group, *p < 0.05.

Our animal experiments suggested that *luteolin* can promote the osteogenic differentiation of BMSCs, and the results of a network pharmacological analysis show that *luteolin* can regulate the PI3K/Akt signalling pathway. Therefore, we further explored the effect of *luteolin* on PI3K/Akt signalling pathway activity. The results of WB showed that the levels of phosphorylated PI3K and Akt (p-PI3K/PI3K and p-Akt/Akt) in BMSCs of the OVX group were significantly lower than those of BMSCs of the sham group. The levels BMSCs of in the *Luteolin-*L group, *Luteolin*-H group and positive drug group were higher than those in BMSCs of the OVX group (**Figures 10A, B**). After BMSCs were cultured with *luteolin* at concentrations of 0.5, 1 and 5 μM for 48 h, the relative expression of phosphorylated PI3K and Akt (p-PI3K/PI3K and p-Akt/Akt) was also significantly higher than that in the DMSO group (**Figures 10C, D**). To verify the role of the PI3K/Akt signalling pathway in the ability of *luteolin* to promote the osteogenic differentiation of BMSCs, we used the PI3K inhibitor LY294002 to inhibit the activity of the PI3K/Akt signalling pathway in BMSCs. The results of WB showed that the p-Akt/Akt expression ratio in BMSCs treated with 20 μM LY294002 was significantly lower than that in the DMSO group, indicating that LY294002 inhibited the activity of the PI3K/Akt signalling pathway in the BMSCs. LY294002 also reduced the protein expression levels of collagen I, osteopontin and RUNX2 in the BMSCs; the protein expression levels of collagen I, osteopontin and RUNX2 in BMSCs cultured with both 20 μM LY294002 and 5 μM *luteolin* were significantly lower than those in BMSCs cultured with 5 μM *luteolin* alone (**Figures 10E, F**), suggesting that inhibiting the activity of the PI3K/Akt signalling pathway reduced the ability of *luteolin* to promote the osteogenic differentiation of BMSCs. The above results suggest that *luteolin* may promote the osteogenic differentiation of BMSCs by enhancing the activity of the PI3K/Akt signalling pathway.

## Discussion

Studies suggest that *luteolin* can reduce bone loss by reducing osteoclast activity and differentiation function (17, 18). Therefore, based on the large number of sources of *luteolin* and its great potential for the treatment of OP, in this study, we combined network pharmacology and experimental verification to explore the mechanism of *luteolin* in the treatment of OP. Through the prediction of *luteolin* and disease-related targets by network pharmacology, 54 potential targets of *luteolin* in OP were screened. PPI network analysis showed that *luteolin* may inhibit OP by regulating TP53, AKT1, JUN, RELA, CASP3, MAPK1, RB1 and CDKN1A. GO enrichment analysis found that the biological processes enriched in the targets of *luteolin* against OP include the response to drugs, negative regulation of apoptotic processes, and positive regulation of nitric oxide biosynthetic processes. The results of KEGG pathway enrichment analysis showed that *luteolin* may play a key role in regulating the PI3K-Akt, TNF, HIF-1, oestrogen and p53 signalling pathways. These results suggest that *luteolin* can regulate the biological processes of OP through multiple targets and pathways. The results of GO enrichment and KEGG pathway enrichment analyses showed that the degree to which the PI3K-Akt signalling pathway is enriched in targets of *luteolin* and OP is high, which may have significance and value for treating OP.

The results of animal experiments show that *luteolin* could reduce bone loss in OVX rats and upregulate the protein expression of collagen I, osteopontin and RUNX2 in BMSCs to promote osteogenic differentiation. *In vitro* cellular studies showed that *luteolin* promoted the osteogenic differentiation of BMSCs by increasing the p-PI3K/PI3K and p-Akt/Akt expression ratios, subsequently enhancing the activity of the PI3K/Akt signalling pathway. One study (19) showed that activation of the PI3K/Akt signalling pathway could stimulate the proliferation and differentiation of osteoblasts and inhibit their apoptosis. In addition, PI3K can stimulate the formation of osteoclast actin filaments and regulate cell chemotaxis, adhesion and diffusion. Inhibiting PI3K expression can reduce bone resorption of mature osteoclasts (19). Luteolin is a flavonoid monomer. Other research into the use of flavonoids for the treatment of OP has revealed that flavonoids, such as *epimedium* and *drynaria*, can regulate the activity balance of osteoblasts and osteoclasts by activating the PI3K/Akt signalling pathway (20, 21). The results of PPI analysis showed that AKT1 and TP53 are key targets of *luteolin* in the treatment of OP. Compared with that in normal mice, bone mass in AKT1- and AKT2-knockout mice are significantly decreased, suggesting that this pathway is related to enhanced bone formation (22). The PI3K/Akt signalling pathway is also negatively regulated by several factors. For example, TP53 can negatively regulate the PI3K/Akt signalling pathway and promote the dephosphorylation of members of the PI3K/Akt signalling pathway through a series of biological effects (23, 24). Studies have shown that the PI3K-Akt signalling pathway can affect bone formation and bone cell survival, thereby controlling BMD balance (25, 26). Based on this information and our network pharmacology and experimental research results, we believe that *luteolin* may enhance the activity of the PI3K-Akt signalling pathway by interfering with target proteins such as AKT1 and TP53 to inhibit OP.

This study has the following limitations, and further theoretical and experimental research is needed in the future. First, public databases are dynamic, so the target data associated with the results of this study may change in the future. Additionally, this study did not comprehensively verify the targets and pathway results, which were theoretically derived. Other pathway and target data that were not verified in this study need to be further studied in future work.

## Conclusions

Based on network pharmacology experiments and an established OP rat model, this study discusses the mechanism of *luteolin* in the treatment of OP, and the PI3K-Akt signalling pathway was selected for further experimental verification. The results show that *luteolin* can reduce bone loss in OVX rats, and upregulate the protein expression of collagen I, osteopontin and RUNX2 in BMSCs, promoting osteogenic differentiation. The possible mechanism by which *luteolin* acts against osteoporosis is the promotion of BMSC osteogenic differentiation *via* PI3K Akt signalling pathway activation regulation. This study provides new insights for the experimental and clinical treatment of OP in the future.

## Data Availability Statement

The original contributions presented in the study are included in the article/**Supplementary Material**. Further inquiries can be directed to the corresponding authors.

## Ethics Statement

The animal study was reviewed and approved by the Experimental Animal Center of Guangdong College of Traditional Chinese Medicine.

## Author Contributions

GL, JZ, LZ, and WY contributed to the concept and design of this study. GL, JZ, YD, YY, ZZ, and RZ performed the computational analyses and performed the *in vivo* experiments. GL, JZ, YD, DZ, YY, and ZZ performed the experimental analysis. GL and JZ drafted the manuscript. All the authors read, revised, and approved the final manuscript.

## Funding

This work was supported by the National Natural Science Foundation of China (No. No.82004383, No. 81974574), the National key research and development program (2021YFC1712804), the Project of Administration of Traditional Chinese Medicine of Guangdong Province (No.20201129, No.20225016, No.20225025), the Project of Guangdong Provincial Department of Finance (No. [2014]157), the Medical Science Research Foundation of Guangdong Province (No.A2020105, No.B2019091), the Science and Technology Planning Project of Guangzhou (No. 202102010273) and the Science and Technology Research Project of Guangdong Provincial Hospital of Chinese Medicine (No.YN2019ML08).

## Conflict of Interest

The authors declare that the research was conducted in the absence of any commercial or financial relationships that could be construed as a potential conflict of interest.

## Publisher’s Note

All claims expressed in this article are solely those of the authors and do not necessarily represent those of their affiliated organizations, or those of the publisher, the editors and the reviewers. Any product that may be evaluated in this article, or claim that may be made by its manufacturer, is not guaranteed or endorsed by the publisher.
